# Lower Blood Pressure Extremes Precede the Recorded Diagnosis of Fibromyalgia by up to Two Decades: A Population-Based Matched Case–Control Study

**DOI:** 10.3390/jcdd13100474

**Published:** 2026-09-22

**Authors:** Eli Magen, Suhail Aamar, Israel Magen, Eugene Merzon, Ilan Green, Avivit Golan-Cohen, Shlomo Vinker, Ariel Israel

**Affiliations:** 1 Adelson School of Medicine, Ariel University, Ariel 4070000, Israel; emarzon@leumit.co.il; 2 Leumit Health Services, Tel Aviv-Yafo 6473817, Israel; igreen@leumit.co.il (I.G.); agolanchoen@leumit.co.il (A.G.-C.); svinker@leumit.co.il (S.V.); aisrael@leumit.co.il (A.I.); 3 Rheumatology Unit, Assuta Ashdod University Medical Center, Ben-Gurion University of the Negev, Ashdod 7747629, Israel; aamars@hadassah.org.il; 4 Urology Department, Assuta Ashdod University Medical Center, Ben-Gurion University of the Negev, Ashdod 7747629, Israel; imagen@leumit.co.il; 5 Department of Family Medicine, Faculty of Medicine, Tel-Aviv University, Tel Aviv-Yafo 6997801, Israel; 6 Department of Epidemiology and Disease Prevention, Gray Faculty of Medical & Health Sciences, Tel Aviv University, Tel Aviv-Yafo 6997801, Israel

**Keywords:** fibromyalgia, blood pressure, hypotension, heart rate, pulse pressure, autonomic nervous system, electronic health records, case–control study

## Abstract

Fibromyalgia (FM) has been linked to autonomic dysregulation, but population-level evidence on blood pressure (BP) in FM is sparse, and whether BP differences are present before diagnosis is unknown. We conducted a population-based case–control study within Leumit Health Services, Israel, comparing 15,869 adults with FM with 79,345 controls matched 5:1 on sex, birth year, and membership start. BP, heart rate (HR), and body temperature were summarized as each individual’s minimum, mean, and maximum values over the full observation period and within a pre-diagnosis window extending up to 20 years before the index date with a 30-day blackout. Over the full period, FM patients had lower minimum systolic BP (99.1 ± 11.1 vs. 102.6 ± 12.3 mmHg; standardized mean difference [SMD] −0.29, 95% CI −0.31 to −0.27), lower minimum diastolic BP and pulse pressure, and higher maximum HR (SMD +0.22), whereas mean systolic BP differed little (SMD −0.08). The lower-tail BP difference was already present before diagnosis (minimum systolic BP −3.27 mmHg, 95% CI −3.51 to −3.04; SMD −0.24), in every interval from 20 years before the index date, and with 90-, 180-, and 365-day blackouts and across diagnostic-criteria eras. FM patients had more BP readings (22.1 vs. 15.3 per patient), and greater measurement intensity amplified the observed difference; nevertheless, the association persisted, attenuated, when measurement opportunity was standardized (first 3, 5, or 10 readings; measurement count strata; patient-level 10th percentile) and after multivariable adjustment for measurement intensity and cardiometabolic and cardiovascular covariates (−1.18 mmHg, 95% CI −1.37 to −0.99). The HR difference was small after adjustment (+0.25 bpm, 95% CI 0.03 to 0.48). Coded hypotensive, orthostatic, and autonomic diagnoses and dispensing of midodrine and fludrocortisone were more frequent in FM before diagnosis. A modest but reproducible lower-tail BP difference therefore precedes the recorded diagnosis of FM by many years. Because symptoms also antedate diagnosis, these data establish precedence over diagnosis rather than over disease onset, and they are compatible with, but do not prove, altered cardiovascular-autonomic regulation.

## 1. Introduction

Fibromyalgia (FM) is a common chronic pain disorder, affecting roughly 2% of adults and predominantly women, defined by widespread pain, fatigue, sleep disturbance, and cognitive symptoms [1,2]. Although it is classified among the rheumatic conditions, a large body of physiological work places dysfunction of the autonomic nervous system near the centre of its pathophysiology [1,2,3,4]. Patients show resting sympathetic predominance with blunted reactivity, abnormal heart rate variability, and a high prevalence of orthostatic intolerance [3,4,5,6,7,8,9].

How this autonomic disturbance manifests in routine cardiovascular measurements has remained surprisingly unclear [3,4]. The clinically focused tilt-table literature, based on samples of a few dozen patients, repeatedly reports exaggerated falls in BP and abnormal heart rate responses to standing, pointing toward a hypotensive tendency [3,4,6,10,11]. Population-level data, by contrast, have focused on whether FM raises the risk of cardiovascular disease and have treated BP as only one covariate among many [12,13]. These two bodies of literature have never been reconciled, and a basic descriptive question remains unanswered: what is the blood pressure of people with fibromyalgia, measured at scale, in ordinary clinical care [3,12]?

A second gap is temporal [3,12]. Because almost all prior studies sampled BP at or after the FM diagnosis, they could not determine whether cardiovascular differences are already present before clinical recognition. Demonstrating a pre-diagnosis pattern would establish precedence over the recorded diagnosis and over FM-specific management, albeit not necessarily over biological or symptomatic disease onset because FM is typically diagnosed after a prolonged symptomatic period [14].

We therefore used a large electronic health record (EHR) cohort to characterize the distribution of BP and related vital signs in FM compared with matched controls and, critically, to determine when any difference becomes detectable relative to diagnosis [3,12,14]. We examined each individual’s minimum, mean, and maximum values, reasoning that an intermittent tendency toward low pressure would be most visible in the lower tail of the distribution. Because an observed minimum depends on how often a person is measured, and because FM patients have more healthcare contact, we performed dedicated analyses of measurement intensity, within-person variability, longer diagnostic blackouts, diagnostic-criteria era, and cardiometabolic and cardiovascular confounding. We hypothesized that FM would be associated with a reproducible lower-tail BP difference, accompanied by a higher heart rate, which is detectable before the recorded diagnosis [12,15,16].

## 2. Materials and Methods

### 2.1. Study Design and Population

We performed a population-based case–control study using the electronic health records of Leumit Health Services (LHS), a nationwide Israeli health-maintenance organization serving approximately 750,000 members with near-complete capture of diagnoses, prescriptions, laboratory results, and vital signs. Eligible cases were LHS members aged 18–90 years with a first recorded diagnosis of FM between January 2002 and December 2023. Diagnoses before 2010 followed the 1990 American College of Rheumatology (ACR) criteria; later diagnoses followed the 2010/2011 criteria and their 2016 revision [17,18,19]. Diagnoses were identified from coded records and extracted through March 2024. The index date was the date of the first FM diagnosis. Members with no recorded vital-sign measurements during the observation period were not included in the vital-sign analyses; no other clinical exclusion criteria were applied at cohort assembly so that confounding by comorbidity and medication could be addressed analytically (Section 2.3) rather than by selection.

Each case was matched to five controls on sex and year of first LHS membership; for every case, the five controls with the closest birth dates were selected without replacement, and each control was assigned the index date of its matched case to synchronize follow-up. The study was approved by the LHS ethics committee (LEU 05-23); informed consent was waived given the retrospective design and use of de-identified data.

### 2.2. Vital-Sign, Comorbidity, and Medication Data

For each participant, we extracted all recorded office BP measurements (automated oscillometric devices), heart rate, body temperature, oxygen saturation, anthropometric measurements, and laboratory values. Before patient-level summaries were calculated, implausible values were excluded using prespecified ranges: systolic BP 60–250 mmHg, diastolic BP 30–150 mmHg, pulse pressure > 10 mmHg, heart rate 30–220 bpm, and body temperature 34–42 °C. For each individual, we calculated the minimum, mean, and maximum systolic BP and diastolic BP; the mean arterial pressure and pulse pressure; the maximum heart rate; and the minimum body temperature. We also recorded, for each individual, the number of BP readings, the number of encounters with a BP reading, and the measurement rate (readings per year of observation). Comorbidities (obesity, smoking, diabetes, dyslipidemia, chronic kidney disease, ischemic heart disease, congestive heart failure, cerebrovascular disease, and hypertension) and medication exposures, including all antihypertensive classes (ATC C02, C03, C07, C08, C09) and drugs used to treat hypotension (midodrine and fludrocortisone), were obtained from coded diagnoses, laboratory data, and dispensing records. Coded diagnoses of orthostatic hypotension (ICD-9 458.0), chronic hypotension (458.1), unspecified hypotension (458.9), autonomic disorder or neuropathy (337.0, 337.00, 337.90), syncope or presyncope (780.2, 780.22, 780.23), and unspecified tachycardia (785.0) were extracted as supportive clinical evidence; standardized orthostatic BP protocols, tilt-table testing, beat-to-beat BP, and heart rate variability were not systematically available in the EHR.

### 2.3. Pre-Diagnosis and Sensitivity Analyses

To establish temporality relative to diagnosis, we repeated the vital-sign analyses using only measurements recorded before the index date, within a window extending up to 20 years earlier, with a 30-day peri-diagnostic blackout so that readings taken during the diagnostic encounter itself were not included. A pattern that persists in this window cannot be a consequence of the FM label, of FM-specific treatment, or of peri-diagnostic care; it does not, however, establish precedence over the onset of symptoms. To characterize the time course directly, we additionally analyzed non-overlapping intervals (−20 to −15, −15 to −10, −10 to −5, −5 to −2, −2 years to −30 days, 0–2, 2–5, and 5–10 years relative to the index date). Robustness to the peri-diagnostic period was assessed with progressively longer 90-, 180-, and 365-day blackouts, and analyses were stratified by diagnostic-criteria era (<2010, ≥2010, 2010–2015, and ≥2016).

Because a patient’s observed minimum depends on the number of available measurements, we (i) compared measurement counts and encounter counts between groups; (ii) repeated the primary analysis using only each participant’s first 3, 5, or 10 chronological readings, which equalizes measurement opportunity; (iii) stratified participants by total pre-diagnosis BP-reading count (2–5, 6–10, 11–20, 21–50, >50); (iv) fitted patient-level linear models adjusting for log-transformed measurement count and measurement rate; and (v) used each participant’s 10th percentile of systolic BP, which is less sensitive than the minimum to a single extreme reading. Within-person standard deviation (SD) and coefficient of variation (CV) of systolic BP, diastolic BP, and heart rate were calculated among participants with at least three eligible pre-diagnosis measurements to examine whether greater variability rather than a downward shift could account for a lower minimum.

Confounding was addressed in two ways. First, a restricted cohort excluded participants with any antihypertensive dispensing or with a diagnosis of ischemic heart disease, congestive heart failure, cerebrovascular disease, or diabetes before the pre-diagnosis cutoff. Second, patient-level multivariable linear models were fitted for pre-diagnosis minimum systolic BP and maximum heart rate. The primary model adjusted for age, sex, body-mass index (BMI) category and within-category BMI, smoking, diabetes, dyslipidemia, renal function (estimated glomerular filtration rate class), antihypertensive exposure, log measurement count, and measurement rate; an extended cardiovascular model additionally included socioeconomic status, ischemic heart disease, congestive heart failure, cerebrovascular disease, chronic kidney disease, hypertension, and beta-blocker exposure.

### 2.4. Statistical Analysis

Continuous variables are summarized as mean ± SD and median [interquartile range, IQR]; categorical variables as counts and percentages. Between-group differences were quantified as mean differences with 95% confidence intervals (CIs), standardized mean differences (SMDs, FM minus control, with 95% CIs), and two-sided Welch *t*-tests; an absolute SMD of 0.10–0.20 was regarded as small and ≥0.20 as small-to-moderate. Binary outcomes were summarized as odds ratios (ORs) with 95% CIs from logistic regression, with exact methods for sparse outcomes. Because cases and controls were matched on sex, birth year, and membership year, these variables are balanced by design; the primary contrasts treated the two groups as independent samples, which is conservative for matched data, and the multivariable models additionally adjusted for the matching variables, age, and sex. Benjamini–Hochberg false discovery rate correction was applied within related analysis families. Analyses used R 4.4. Effect sizes and their consistency across sensitivity analyses were interpreted alongside statistical significance, because with approximately 95,000 participants even trivial differences reach conventional significance thresholds.

## 3. Results

### 3.1. Cohort Characteristics and Measurement Intensity

The cohort comprised 15,869 FM patients and 79,345 matched controls. By design, the groups were identical in sex (82.1% female) and mean age (48.5 ± 13.5 years). FM patients carried a heavier burden of cardiovascular risk factors: higher BMI (28.3 ± 6.0 vs. 27.6 ± 5.8 kg/m^2^; obesity 35.4% vs. 29.9%, OR 1.28, 95% CI 1.24–1.33), more current smoking (22.4% vs. 17.5%; OR 1.36, 95% CI 1.30–1.42), more dyslipidemia (33.2% vs. 28.2%; OR 1.27, 95% CI 1.22–1.32), higher triglycerides and lower HDL cholesterol, a higher prevalence of diabetes (11.7% vs. 9.9%; OR 1.21, 95% CI 1.15–1.28), and more coded hypertension (24.6% vs. 21.9%; OR 1.16, 95% CI 1.12–1.21), whereas coded chronic kidney disease did not differ (0.71% vs. 0.65%; OR 1.09, 95% CI 0.88–1.35) (Table 1). FM patients also had more pre-diagnosis BP readings than controls (mean 22.1 vs. 15.3 per patient; median 12 vs. 8; difference +6.73, 95% CI 6.19 to 7.27; SMD +0.25) and more encounters with a BP reading (21.4 vs. 15.0; median 12 vs. 7; SMD +0.25) (Table 2).

### 3.2. Lower Blood Pressure Extremes and a Higher Maximum Heart Rate over the Full Observation Period

Across the full observation period, FM patients showed a consistent difference concentrated in the lower tail of the BP distribution (Table 3, Figure 1). Minimum systolic BP was lower in FM (99.13 ± 11.13 vs. 102.65 ± 12.30 mmHg; difference −3.51 mmHg, 95% CI −3.71 to −3.32; SMD −0.290, 95% CI −0.307 to −0.273), as were minimum diastolic BP (59.54 ± 7.98 vs. 61.72 ± 8.45 mmHg; SMD −0.261), minimum mean arterial pressure (74.41 ± 8.19 vs. 76.88 ± 8.94 mmHg; SMD −0.281), and minimum pulse pressure (29.33 ± 7.41 vs. 31.79 ± 8.03 mmHg; SMD −0.310). Maximum heart rate was higher (96.80 ± 13.47 vs. 93.85 ± 13.68 bpm; difference +2.95 bpm; SMD +0.216), and minimum body temperature was lower (36.52 ± 0.61 vs. 36.67 ± 0.70 °C; SMD −0.225). Pain intensity, recorded on a visual analogue scale in the subset with documented pain scores, was, as expected, higher in FM (per-patient median 6.54 ± 2.92 vs. 5.04 ± 3.36; difference +1.50, 95% CI 1.14 to 1.87; SMD +0.46). This pattern is compatible with altered cardiovascular-autonomic regulation, but routine EHR vital signs do not measure stroke volume, baroreflex function, or orthostatic responses.

Mean systolic BP showed a much smaller difference (122.51 ± 12.45 vs. 123.61 ± 13.61 mmHg; SMD −0.082) than the minimum, whereas maximum systolic BP was slightly higher in FM (150.82 ± 24.16 vs. 148.84 ± 25.16 mmHg; SMD +0.079). The combination of a pronounced lower-tail difference with a small shift in the mean and opposite movement of the maximum indicates a broader BP distribution in FM rather than a uniform downward displacement of all readings.

### 3.3. The Lower-Tail Difference Precedes the Recorded Diagnosis

When the analysis was restricted to measurements recorded before the index date, the same pattern was present (Table 3, Figure 1). Pre-diagnosis minimum systolic BP was lower in future FM patients (102.55 ± 12.88 vs. 105.82 ± 13.83 mmHg; difference −3.27 mmHg, 95% CI −3.51 to −3.04; SMD −0.239), with concordant reductions in minimum diastolic BP (SMD −0.209), mean arterial pressure (SMD −0.230), and pulse pressure (SMD −0.261), a higher maximum heart rate (89.51 ± 13.74 vs. 87.30 ± 13.50 bpm; difference +2.21 bpm; SMD +0.163), and a lower minimum body temperature (SMD −0.186). Pain scores were also already higher before the diagnosis was recorded (6.87 ± 2.57 vs. 5.28 ± 3.28; SMD +0.51, 95% CI 0.16 to 0.85; *p* = 0.006), indicating that core FM symptomatology, and not only the cardiovascular pattern, antedated the label.

The time course analysis located the BP difference in the earliest available interval (Figure 2; Supplementary Table S2). At −20 to −15 years, minimum systolic BP was 2.71 mmHg lower in FM (95% CI −3.28 to −2.14; SMD −0.186) and minimum pulse pressure 1.63 mmHg lower (95% CI −2.00 to −1.27; SMD −0.177), whereas maximum heart rate was only slightly higher (+0.67 bpm, 95% CI 0.13 to 1.21; SMD +0.062; *p* = 0.015). The BP difference increased modestly toward the index date (minimum systolic BP −3.26 mmHg at −5 to −2 years; SMD −0.226) and remained of similar size after diagnosis (−3.14 to −3.37 mmHg), without an abrupt emergence around the index date. The heart rate difference showed a clearer gradient, rising from SMD +0.062 at −20 to −15 years to +0.132 in the two years before diagnosis and peaking at +0.172 in the two years after it.

### 3.4. Robustness to Measurement Intensity, and Within-Person Variability

Because FM patients were measured more often, we examined whether the lower minimum could be a product of greater measurement opportunity (Table 3, Figure 3). Greater measurement intensity did amplify the observed difference: the minimum systolic BP SMD rose from −0.087 (−1.18 mmHg) among participants with only 2–5 readings to −0.263 (−2.83 mmHg) among those with more than 50 readings. However, the difference was present in every measurement count stratum, persisted when each participant was limited to the first 3, 5, or 10 chronological readings (−1.66, −1.86, and −2.05 mmHg; SMD −0.110, −0.130, and −0.152), remained after adjustment for log measurement count and measurement rate (−1.95 mmHg, 95% CI −2.19 to −1.72), and was reproduced with the patient-level 10th percentile of systolic BP (109.01 vs. 111.03 mmHg; −2.01 mmHg, 95% CI −2.23 to −1.80; SMD −0.158). Measurement frequency bias therefore inflates the crude effect size by roughly one-third to one-half but does not produce it.

The maximum heart rate difference was considerably more sensitive to measurement opportunity. Limited to the first 3, 5, or 10 readings, the difference was +0.38, +0.64, and +1.04 bpm (SMD +0.034, +0.055, and +0.085), and after adjustment for measurement count and rate, it was +0.62 bpm (95% CI 0.39 to 0.86). A substantial part of the crude heart rate difference is thus attributable to the greater number of opportunities to record a high value.

Within-person variability was slightly greater in FM (Supplementary Table S1): pre-diagnosis systolic BP SD 11.25 vs. 11.07 mmHg (SMD +0.038) and CV 0.0927 vs. 0.0902 (SMD +0.077); the corresponding diastolic BP SMDs were +0.075 and +0.087, and heart rate SMDs +0.057 and +0.058. These differences are small relative to the lower-tail displacement; greater variability may contribute to, but does not by itself appear sufficient to explain, the persistent lower minimum.

### 3.5. Sensitivity to Longer Blackouts and to Diagnostic-Criteria Era

Extending the peri-diagnostic blackout had no material effect (Supplementary Table S3): pre-diagnosis minimum systolic BP SMDs were −0.239, −0.238, −0.241, and −0.241 with 30-, 90-, 180-, and 365-day blackouts, minimum pulse pressure SMDs −0.261 to −0.257, and maximum heart rate SMDs +0.163 to +0.153. The associations were also consistent across diagnostic eras: the minimum systolic BP SMD was −0.287 for cases diagnosed before 2010 under the 1990 ACR criteria, −0.245 from 2010 onward, −0.265 for 2010–2015, and −0.238 from 2016 onward; the maximum heart rate SMD ranged from +0.127 (<2010) to +0.209 (≥2016).

### 3.6. Restricted Cohort and Multivariable Adjustment

In the restricted cohort of 8107 FM patients and 43,634 controls without antihypertensive exposure or major cardiovascular or diabetic comorbidity, pre-diagnosis minimum systolic BP remained lower in FM (100.78 vs. 103.76 mmHg; −2.98 mmHg, 95% CI −3.28 to −2.68; SMD −0.226), with concordant differences in minimum diastolic BP (SMD −0.192), mean arterial pressure (SMD −0.214), and pulse pressure (SMD −0.242), and a higher maximum heart rate (+1.89 bpm; SMD +0.142) (Supplementary Table S4). In the primary multivariable model, FM was associated with a 1.44 mmHg lower minimum systolic BP (95% CI −1.64 to −1.25; *p* < 0.001) and a 0.30 bpm higher maximum heart rate (95% CI 0.08 to 0.53; *p* = 0.008); in the extended cardiovascular model, the estimates were −1.18 mmHg (95% CI −1.37 to −0.99; *p* < 0.001) and +0.25 bpm (95% CI 0.03 to 0.48; *p* = 0.027). Adjustment for measurement intensity and confounders therefore attenuated the crude BP difference by roughly two-thirds but left a precise, non-null estimate, whereas the adjusted heart rate difference, although statistically distinguishable from zero, was clinically negligible.

### 3.7. Discordance Between Blood Pressure and Metabolic Risk

The lower-tail BP difference coexisted with a more adverse metabolic profile (Table 1, Figure 4). Despite more obesity, smoking, diabetes, and dyslipidemia, FM patients had lower minimum pressures and no excess of measured grade-2 hypertension (12.5% vs. 13.3%; OR 0.93, 95% CI 0.88–0.98), even though a coded diagnosis of hypertension was somewhat more frequent (OR 1.16), and the distribution of BP categories at the index date was nearly identical between groups. This discordance persisted after adjustment for the metabolic covariates (Section 3.6). It is compatible with altered cardiovascular-autonomic regulation but does not establish that BP in FM is governed by a fixed autonomic set point, nor that conventional cardiovascular risk equations are miscalibrated in FM.

### 3.8. Supportive Pharmacological and Diagnostic Evidence

Drugs used specifically to raise blood pressure were dispensed more often to FM patients (Table 2, Figure 5). Over the full observation period, midodrine was dispensed to 65 of 15,869 FM patients versus 92 of 79,345 controls (OR 3.54, 95% CI 2.54–4.92) and fludrocortisone to 22 versus 52 (OR 2.12, 95% CI 1.22–3.55; *p* = 0.005). Before diagnosis, midodrine was dispensed to 41 versus 34 (OR 6.04, 95% CI 3.74–9.82) and fludrocortisone to 17 versus 33 (OR 2.58, 95% CI 1.35–4.76; *p* = 0.003). Absolute numbers are small and the intervals correspondingly wide. Coded diagnoses recorded in the pre-diagnosis window showed the same direction of association (Supplementary Table S5): orthostatic hypotension (0.50% vs. 0.27%; OR 1.85, 95% CI 1.41–2.41), chronic hypotension (OR 3.39, 95% CI 2.26–5.05), unspecified hypotension (OR 1.79, 95% CI 1.58–2.03), autonomic disorder or neuropathy (OR 4.80, 95% CI 3.38–6.81), syncope or presyncope (11.42% vs. 6.43%; OR 1.88, 95% CI 1.77–1.99), and unspecified tachycardia (OR 1.63, 95% CI 1.45–1.81). These are coded clinical diagnoses and prescriptions, not standardized autonomic tests; they indicate that clinicians recognized and treated hypotensive and orthostatic symptoms more often in future FM patients, but the same symptoms that lead to FM evaluation also lead to orthostatic assessment and empirical prescribing, so these findings are supportive rather than independent evidence.

## 4. Discussion

In a cohort of nearly 95,000 individuals, FM was associated with a reproducible cardiovascular pattern characterized chiefly by lower minimum BP and pulse pressure, accompanied by a smaller higher heart rate signal and lower body temperature. Lower BP extremes were detectable in routine records in every interval from 20 years before the recorded diagnosis, were stable with peri-diagnostic blackouts of up to one year and across diagnostic-criteria eras, and persisted, attenuated, when measurement opportunity was standardized, in a restricted cohort free of antihypertensive exposure and major cardiovascular comorbidity, and after multivariable adjustment. The adjusted estimate of roughly 1.2–1.5 mmHg for the minimum systolic BP is modest in absolute terms but precise and consistent in direction across every analysis. The heart rate component is weaker: after adjustment for measurement intensity and confounders, it shrinks to a fraction of a beat per minute. The data therefore support a modest, longstanding lower-tail BP difference that precedes the recorded diagnosis of FM, rather than a pronounced hypotensive and tachycardic state; and because pain and symptom burden also antedate the diagnosis, they establish precedence over diagnosis, not over disease onset.

### 4.1. Reconciling a Fragmented Literature

Our results help reconcile apparently discordant literature [3,12]. Physiological studies have suggested orthostatic intolerance and altered cardiovascular regulation in FM, whereas database studies based mainly on mean BP have found little difference [3,4,6,10,11,12]. In the present data, the mean BP shift was small, while the lower tail was displaced downward and maximum systolic BP was slightly higher; within-person variability was also slightly greater in FM. The reconciliation is therefore partly methodological: an intermittent tendency to low pressure is averaged away in the mean but preserved in the minimum, so that distribution-based summaries reveal a signal that point estimates conceal. The revised interpretation is not that a single fixed BP set point shifts downward in FM but that FM is associated with a somewhat broader BP distribution and a persistent lower-tail displacement that remains after accounting for measurement opportunity. Because the variability differences were small (SMD 0.04–0.09), greater variability alone appears insufficient to account for the lower minimum, but it cannot be excluded as a contributor, and a design with standardized, equally spaced measurements would be needed to separate the two.

### 4.2. Precedence over Diagnosis, Not Necessarily over Disease Onset

The most distinctive finding is the presence of the BP difference far upstream of the recorded diagnosis [3,4,14]. The −20- to −15-year interval shows that lower minimum systolic BP and pulse pressure are present long before clinical recognition, and the blackout analyses exclude an artefact of peri-diagnostic care. The diagnosis date is not, however, the date of disease onset. FM is characteristically recognized after a prolonged symptomatic period [14], and in our own data, pain scores were already elevated in the pre-diagnosis window. The principal competing interpretation is therefore reverse causation by prodromal or evolving illness: the BP pattern may be an early manifestation of developing FM, a consequence of the symptoms, deconditioning, sleep disturbance, or medication use that accompany the diagnostic delay, or a marker of the clinical trajectory that leads a patient toward eventual diagnosis. The time course offers some, but not decisive, discrimination. The BP difference was of similar size at −20 to −15 years and in the years immediately before diagnosis, with only a modest steepening, which sits more comfortably with a longstanding trait than with a process that begins shortly before recognition; the heart rate difference, by contrast, rose progressively toward the index date and peaked after it, a pattern more consistent with an evolving, symptom-related process. We therefore describe the BP finding as pre-diagnostic rather than pre-clinical, and as compatible with, but not demonstrating, an antecedent cardiovascular trait.

### 4.3. Possible Mechanisms

Several mechanisms could produce a lower BP floor with a modestly higher heart rate ceiling, and the present data cannot distinguish between them. Small studies using tilt-table and beat-to-beat recordings have documented exaggerated orthostatic falls in BP, orthostatic tachycardia, and reduced orthostatic tolerance in FM [8,10,11] and have linked these to blunted baroreflex sensitivity across its cardiac, vasomotor, and myocardial branches [6], to a resting shift in sympathovagal balance toward sympathetic predominance with diminished reactivity to challenge [3,4,5,9], and to autonomic small-fibre pathology [4,7]. A blunted baroreflex would permit larger transient falls in pressure during ordinary daily perturbations, while preserved or increased cardiac sympathetic drive would raise the heart rate recorded at symptomatic or stressful encounters; either could lower the observed minimum without shifting the mean. Reduced stroke volume with compensatory chronotropy is one way to read the narrowed pulse pressure, but stroke volume was not measured. Central pain–autonomic coupling, sleep disturbance, deconditioning, and medication effects offer non-exclusive alternatives, and the slightly lower body temperature may reflect peripheral vasomotor dysregulation [20]. These are hypotheses that the EHR cannot test; they identify what a prospective study with standardized orthostatic BP, heart rate variability, and baroreflex measurements in the pre-diagnosis population should examine.

### 4.4. Discordance from Metabolic Risk and Clinical Implications

FM patients carried more obesity, smoking, diabetes, and dyslipidemia yet did not show the higher measured BP that these factors predict [12,15,16], and the lower-tail difference persisted after adjustment for them. This discordance is compatible with a cardiovascular-regulatory influence on BP in FM, but the study did not test the calibration of cardiovascular risk equations in FM, and we do not claim that BP-weighted scores misestimate risk in this population [21]. Two practical points nevertheless follow. First, a normal or low office BP in a patient with FM does not exclude an adverse metabolic profile, which should be assessed on its own terms. Second, the coexistence of a lower-tail BP tendency, more frequent coded orthostatic and hypotensive diagnoses, and more frequent exposure to antihypertensive and other BP-lowering drugs supports attention to orthostatic symptoms and medication review in symptomatic patients [22,23]; the study did not evaluate the outcomes of such review.

### 4.5. Strengths and Limitations

Strengths include the large matched cohort, long longitudinal depth, repeated routinely collected vital signs, direct analysis of the BP distribution, and an extensive set of sensitivity analyses addressing measurement intensity, variability, blackout length, diagnostic era, and confounding. Several limitations remain. Office oscillometric measurements were obtained in routine care rather than under standardized conditions, and the clinical context of each reading (acute illness, dehydration, and symptomatic visits) could not be harmonized; because such contexts are more frequent in FM, they may contribute to a lower recorded minimum. FM patients were measured more often, and greater measurement intensity amplified the observed difference; the analyses that equalize or model measurement opportunity show that the association is not produced by measurement frequency, but they also show that the crude effect size overstates it. Within-person BP variability was slightly greater in FM and may contribute to the more extreme lower tail. Standardized orthostatic measurements, tilt-table testing, beat-to-beat BP, and heart rate variability were not available, so autonomic mechanisms are inferred rather than measured, and the coded orthostatic, hypotensive, and autonomic diagnoses we report are subject to ascertainment and indication bias. The diagnosis date does not mark disease onset, leaving reverse causation by evolving or prodromal illness possible, as discussed above. Our group comparisons treated matched cases and controls as independent samples, which is conservative, and the multivariable models adjusted for the matching variables; residual confounding by unmeasured comorbidity, psychotropic and analgesic medication, physical activity, or body composition cannot be excluded. Finally, the cohort derives from a single Israeli health system, and external replication is desirable.

## 5. Conclusions

Lower blood pressure extremes are reproducibly associated with a future diagnosis of fibromyalgia and are detectable in routine records up to two decades before that diagnosis. The association is modest after accounting for measurement intensity and cardiometabolic and cardiovascular confounding, but it is precise, directionally consistent across every sensitivity analysis, and stable with longer peri-diagnostic blackouts and across diagnostic-criteria eras; the accompanying heart rate difference is substantially weaker after adjustment. These findings establish temporal precedence over the recorded diagnosis, not over disease onset, and are compatible with, but do not prove, altered cardiovascular-autonomic regulation in fibromyalgia. Prospective studies with standardized orthostatic, beat-to-beat, and heart rate variability measurements in people with early or prodromal symptoms are needed to define the underlying physiology and its clinical relevance.

## Figures and Tables

**Figure 1:**
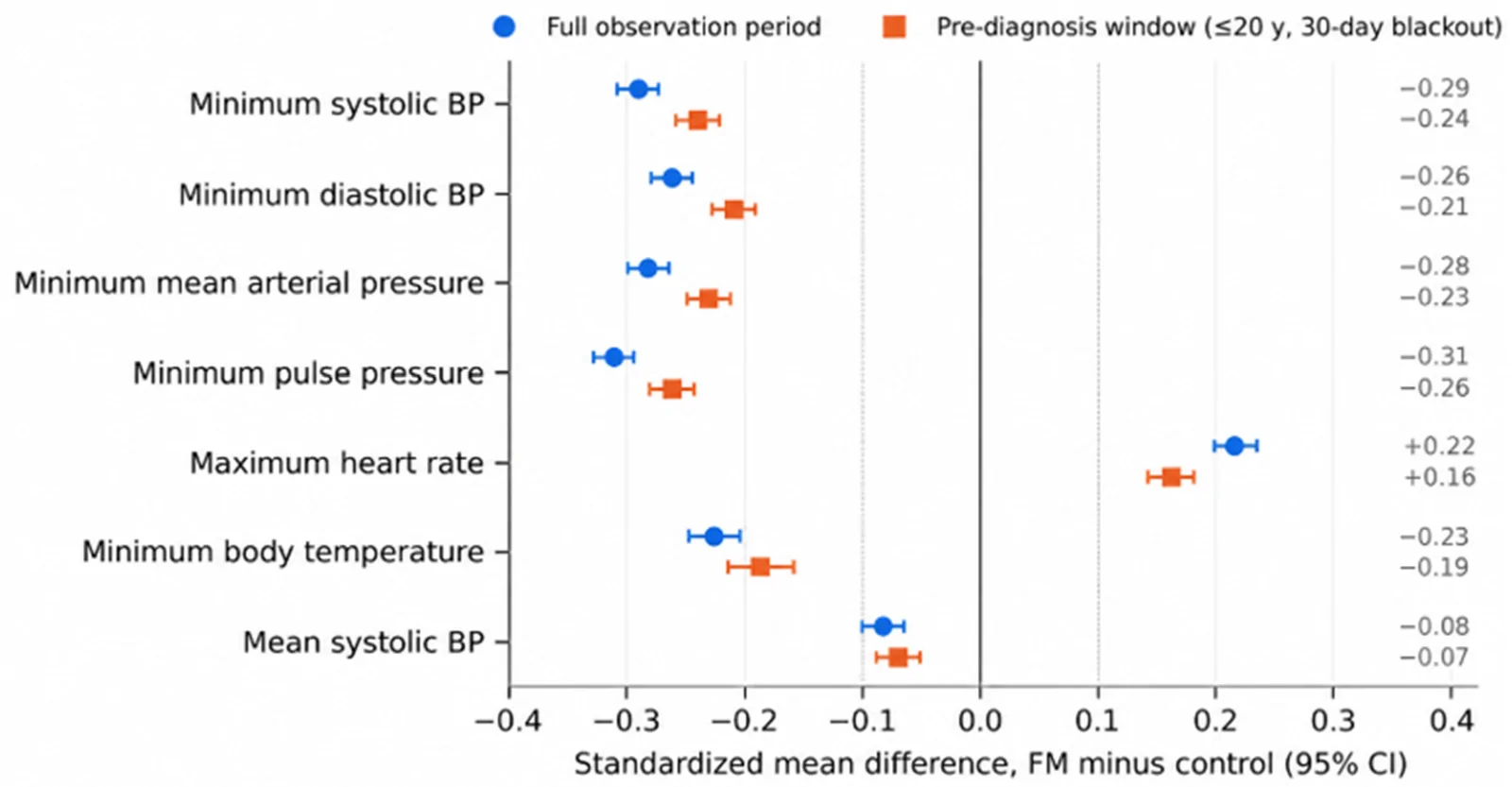
Standardized mean differences (FM minus control) with 95% confidence intervals for the principal vital-sign measures over the full observation period (circles) and within the pre-diagnosis window (up to 20 years before the index date, 30-day blackout; squares). Dotted lines mark SMD ±0.10. Negative values indicate lower values in FM.

**Figure 2:**
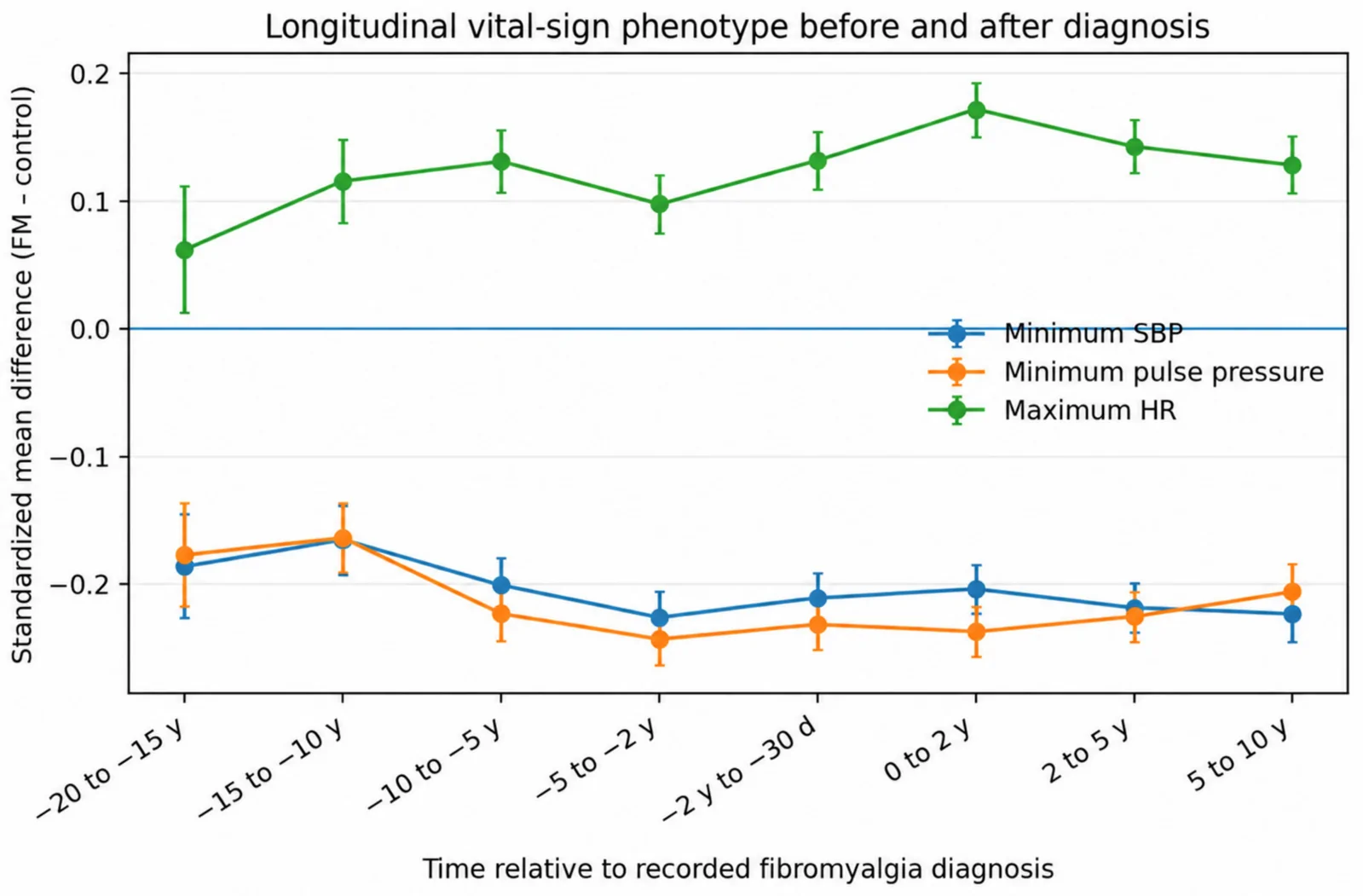
Longitudinal standardized mean differences (FM minus control) for minimum systolic BP, minimum pulse pressure, and maximum heart rate in non-overlapping intervals relative to the recorded fibromyalgia diagnosis. Error bars indicate 95% confidence intervals. Negative values indicate lower values in FM.

**Figure 3:**
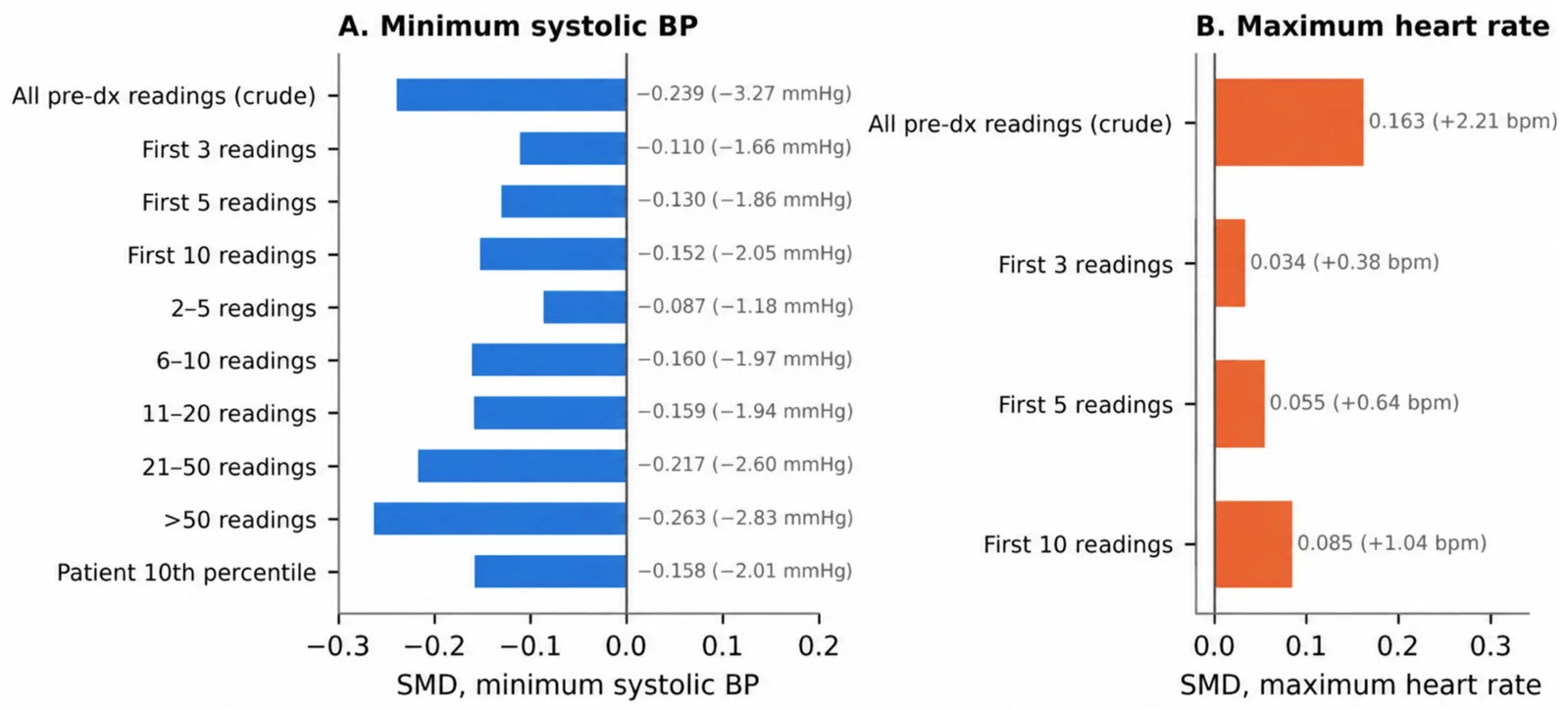
Robustness of the pre-diagnosis differences to measurement opportunity. (**A**) Standardized mean differences (FM minus control) in minimum systolic BP for the crude analysis, the first 3, 5, and 10 chronological readings, strata of total BP-reading count, and the patient-level 10th percentile of systolic BP; the absolute difference in mmHg is shown beside each bar. (**B**) The corresponding analyses for maximum heart rate. Numerical values, confidence intervals, and the count- and rate-adjusted models are given in Table 3 and Supplementary Table S7.

**Figure 4:**
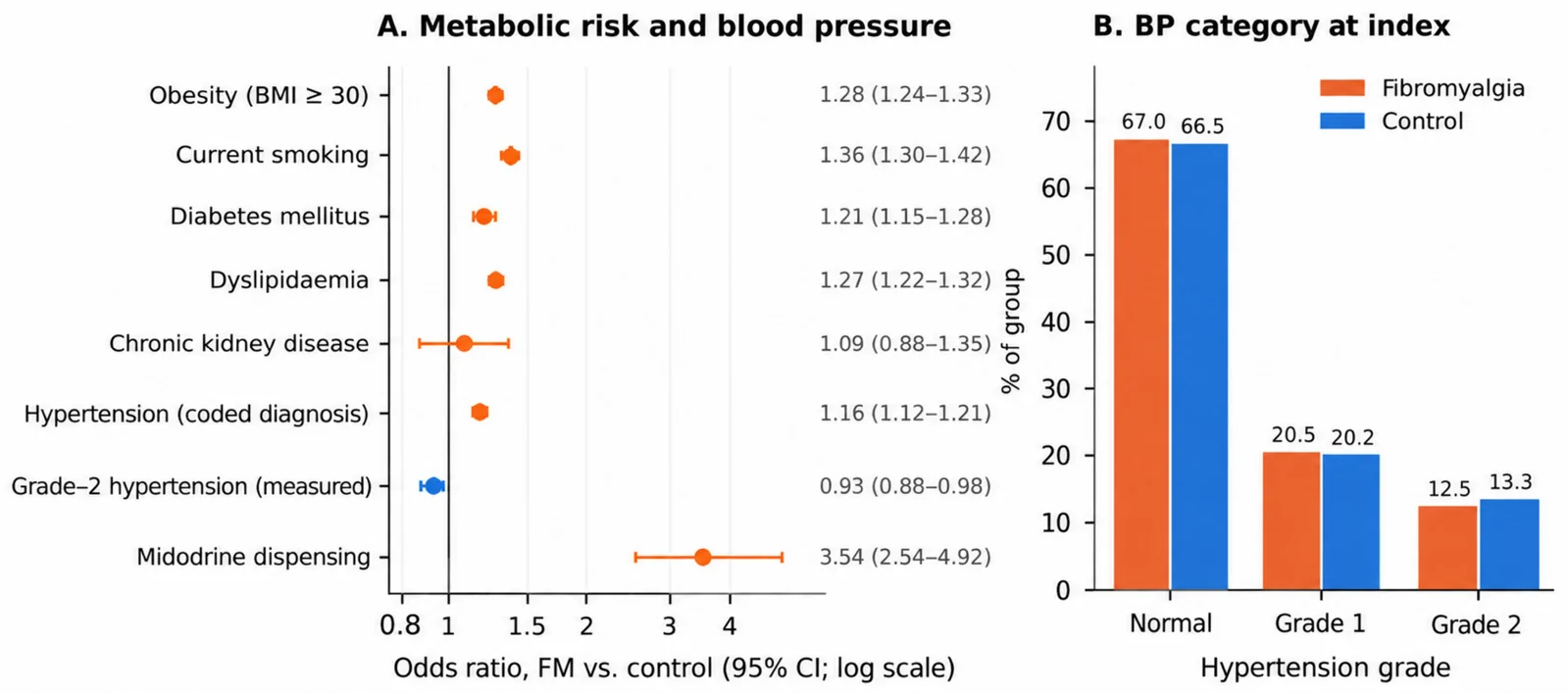
Discordance between metabolic risk burden and measured blood pressure. (**A**) Odds ratios (FM vs. control) with 95% confidence intervals for metabolic risk factors, coded hypertension, and chronic kidney disease at the index date, alongside measured grade-2 hypertension and midodrine dispensing over the full period. (**B**) Blood pressure category at the index date (normal, grade-1 and grade-2 hypertension by measured BP). The figure is descriptive and does not by itself establish the mechanism responsible for the lower-tail BP difference.

**Figure 5:**
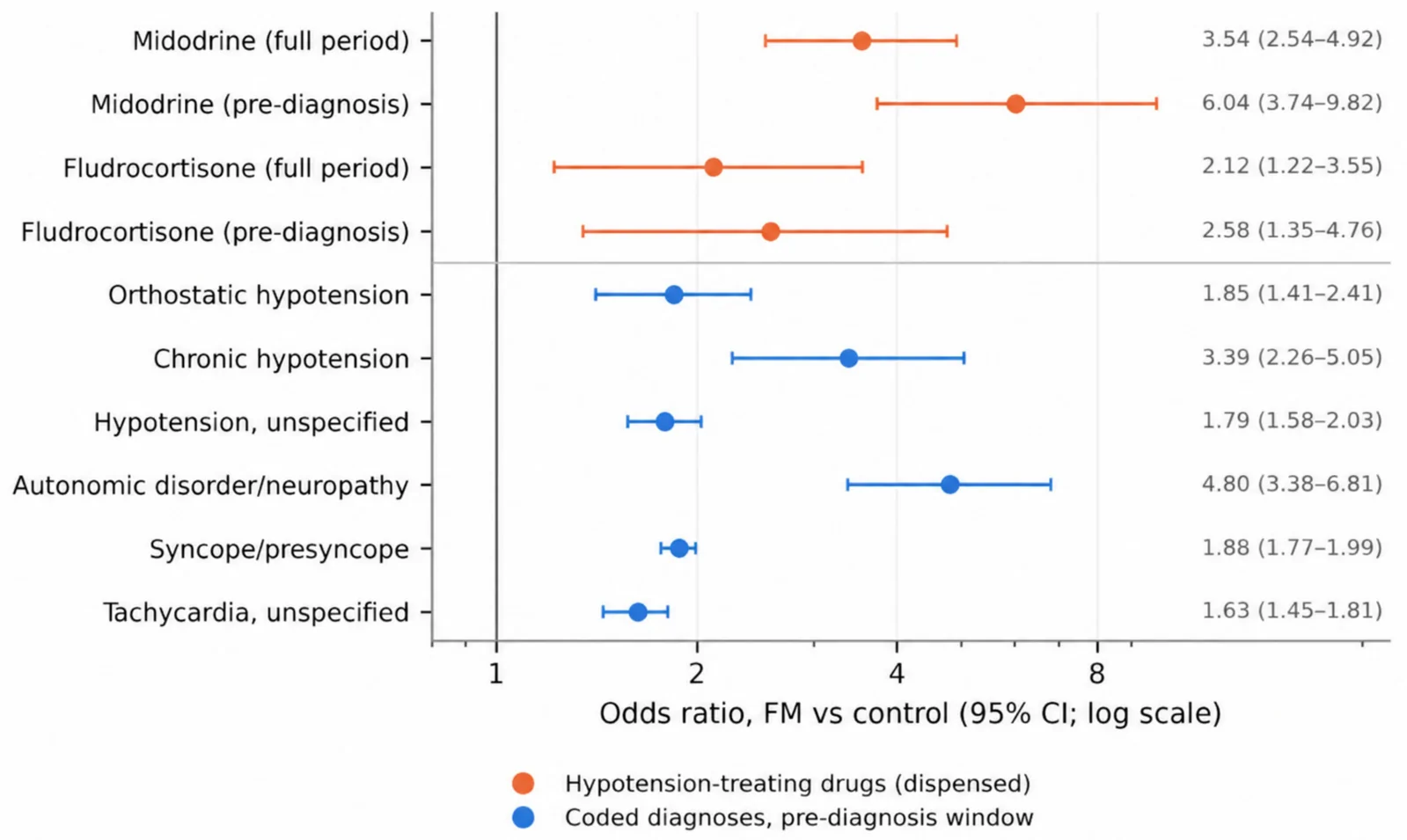
Supportive pharmacological and diagnostic evidence. Odds ratios (FM vs. control) with 95% confidence intervals for dispensing of midodrine and fludrocortisone over the full observation period and in the pre-diagnosis window, and for coded hypotensive, orthostatic, autonomic, syncopal, and tachycardic diagnoses recorded in the pre-diagnosis window. Log scale.

**Table 1:** Metabolic risk factors, measured hypertension, and blood pressure-related medication use.

Variable	FM, *n* (%)	Control, *n* (%)	OR (95% CI)	*p*
Metabolic risk and hypertension at the index date				
Obesity (BMI ≥ 30 kg/m^2^)	5544 (35.4%)	23,101 (29.9%)	1.28 (1.24–1.33)	<0.001
Current smoking	3551 (22.4%)	13,892 (17.5%)	1.36 (1.30–1.42)	<0.001
Diabetes mellitus	1860 (11.7%)	7846 (9.9%)	1.21 (1.15–1.28)	<0.001
Dyslipidemia	5275 (33.2%)	22,370 (28.2%)	1.27 (1.22–1.32)	<0.001
Chronic kidney disease	112 (0.71%)	512 (0.65%)	1.09 (0.88–1.35)	0.39
Hypertension (coded diagnosis)	3897 (24.6%)	17,354 (21.9%)	1.16 (1.12–1.21)	<0.001
Grade-1 hypertension (measured)	3243 (20.5%)	15,832 (20.2%)	1.02 (0.97–1.06)	0.44
Grade-2 hypertension (measured)	1977 (12.5%)	10,416 (13.3%)	0.93 (0.88–0.98)	0.006
Hypotension-treating drugs, full period				
Midodrine	65 (0.41%)	92 (0.12%)	3.54 (2.54–4.92)	<0.001
Fludrocortisone	22 (0.14%)	52 (0.07%)	2.12 (1.22–3.55)	0.005
Hypotension-treating drugs, pre-diagnosis window				
Midodrine	41 (0.26%)	34 (0.04%)	6.04 (3.74–9.82)	<0.001
Fludrocortisone	17 (0.11%)	33 (0.04%)	2.58 (1.35–4.76)	0.003
Any antihypertensive (ATC C02/C03/C07/C08/C09)				
Full period	8836 (55.68%)	36,766 (46.34%)	1.45 (1.41–1.51)	<0.001
Pre-diagnosis window	5503 (34.68%)	22,687 (28.59%)	1.33 (1.28–1.37)	<0.001

OR, odds ratio (FM vs. control). Metabolic variables, coded diagnoses and measured BP category are ascertained at the index date; percentages are of the full cohort (15,869 FM; 79,345 controls), with BMI missing in 1.3% of FM patients and 2.8% of controls and BP in 0.3% and 1.4%. Measured hypertension grades follow the BP category recorded at the index date. *p*-values for drug dispensing are from Fisher’s exact test. Pre-diagnosis dispensing covers 20 years before the index date to 30 days before it. Antihypertensive classes are shown individually in Supplementary Table S5.

**Table 2:** Measurement intensity and robustness of the pre-diagnosis minimum-systolic-BP and maximum heart rate differences to measurement opportunity and confounding.

Analysis	FM	Control	FM − Control Difference (95% CI)	SMD	*p*
Measurement intensity					
BP readings per patient, mean (median)	22.1 (12)	15.3 (8)	+6.73 (6.19 to 7.27)	+0.248	<0.001
Encounters with a BP reading, mean (median)	21.4 (12)	15.0 (7)	+6.45 (5.92 to 6.97)	+0.245	<0.001
Minimum systolic BP (mmHg)					
All pre-diagnosis readings (crude)	102.55	105.82	−3.27 (−3.51 to −3.04)	−0.239	<0.001
First 3 readings	—	—	−1.66 (−1.94 to −1.38)	−0.110	<0.001
First 5 readings	—	—	−1.86 (−2.14 to −1.58)	−0.130	<0.001
First 10 readings	—	—	−2.05 (−2.36 to −1.75)	−0.152	<0.001
Adjusted for log measurement count and rate	—	—	−1.95 (−2.19 to −1.72)	—	<0.001
Patient-level 10th percentile of systolic BP	109.01	111.03	−2.01 (−2.23 to −1.80)	−0.158	<0.001
Restricted cohort ^a^	100.78	103.76	−2.98 (−3.28 to −2.68)	−0.226	<0.001
Multivariable model, primary ^b^	—	—	−1.44 (−1.64 to −1.25)	—	<0.001
Multivariable model, extended cardiovascular ^c^	—	—	−1.18 (−1.37 to −0.99)	—	<0.001
Maximum heart rate (bpm)					
All pre-diagnosis readings (crude)	89.51	87.30	+2.21 (1.95 to 2.47)	+0.163	<0.001
First 3 readings	—	—	+0.38 (0.14 to 0.63)	+0.034	0.002
First 5 readings	—	—	+0.64 (0.35 to 0.93)	+0.055	<0.001
First 10 readings	—	—	+1.04 (0.65 to 1.43)	+0.085	<0.001
Adjusted for log measurement count and rate	—	—	+0.62 (0.39 to 0.86)	—	<0.001
Restricted cohort ^a^	87.85	85.96	+1.89 (1.55 to 2.24)	+0.142	<0.001
Multivariable model, primary ^b^	—	—	+0.30 (0.08 to 0.53)	—	0.008
Multivariable model, extended cardiovascular ^c^	—	—	+0.25 (0.03 to 0.48)	—	0.027

Measurement count and multivariable models are patient-level linear models; negative differences indicate lower values in FM. Group sizes for each analysis and the measurement count strata are given in Supplementary Table S7. ^a^ Participants with no antihypertensive dispensing and no ischemic heart disease, congestive heart failure, cerebrovascular disease, or diabetes before the pre-diagnosis cutoff (8107 FM; 43,634 controls). ^b^ Adjusted for age, sex, BMI category and within-category BMI, smoking, diabetes, dyslipidemia, renal function class, antihypertensive exposure, log measurement count, and measurement rate. ^c^ Additionally adjusted for socioeconomic status, ischemic heart disease, congestive heart failure, cerebrovascular disease, chronic kidney disease, hypertension, and beta-blocker exposure.

**Table 3:** Vital-sign differences between fibromyalgia patients and matched controls over the full observation period and in the pre-diagnosis window (up to 20 years before the index date, 30-day blackout).

Measure (Per-Patient Statistic)	Full Period: FM	Full Period: Control	Full Period: SMD (95% CI)	Pre-Diagnosis: FM vs. Control	Pre-Diagnosis: SMD (95% CI)	*p* (Full; Pre-dx)
Minimum systolic BP (mmHg)	99.13 ± 11.13	102.65 ± 12.30	−0.290 (−0.307 to −0.273)	102.55 ± 12.88 vs. 105.82 ± 13.83	−0.239 (−0.258 to −0.221)	<0.001; <0.001
Minimum diastolic BP (mmHg)	59.54 ± 7.98	61.72 ± 8.45	−0.261 (−0.278 to −0.244)	63.12 ± 8.75 vs. 65.03 ± 9.23	−0.209 (−0.227 to −0.191)	<0.001; <0.001
Minimum mean arterial pressure (mmHg)	74.41 ± 8.19	76.88 ± 8.94	−0.281 (−0.298 to −0.264)	77.37 ± 9.36 vs. 79.64 ± 10.00	−0.230 (−0.248 to −0.212)	<0.001; <0.001
Minimum pulse pressure (mmHg)	29.33 ± 7.41	31.79 ± 8.03	−0.310 (−0.327 to −0.293)	31.81 ± 8.53 vs. 34.14 ± 9.04	−0.261 (−0.279 to −0.242)	<0.001; <0.001
Maximum heart rate (bpm)	96.80 ± 13.47	93.85 ± 13.68	+0.216 (+0.199 to +0.234)	89.51 ± 13.74 vs. 87.30 ± 13.50	+0.163 (+0.144 to +0.182)	<0.001; <0.001
Minimum body temperature (°C)	36.52 ± 0.61	36.67 ± 0.70	−0.225 (−0.246 to −0.203)	36.73 ± 0.73 vs. 36.88 ± 0.80	−0.186 (−0.213 to −0.158)	<0.001; <0.001
Mean systolic BP (mmHg)	122.51 ± 12.45	123.61 ± 13.61	−0.082 (−0.100 to −0.065)	120.13 ± 12.92 vs. 121.09 ± 13.95	−0.069 (−0.087 to −0.051)	<0.001; <0.001
Pain, visual analogue scale (0–10) ^a^	6.54 ± 2.92	5.04 ± 3.36	+0.455 (+0.335 to +0.575)	6.87 ± 2.57 vs. 5.28 ± 3.28	+0.506 (+0.162 to +0.850)	<0.001; 0.006

Values are per-patient summaries (mean ± SD). SMD, standardized mean difference (FM minus control); negative values indicate lower values in FM. Numbers of participants with each measure, median [IQR] values, and mean differences with 95% CIs are provided in Supplementary Table S6. ^a^ Per-patient median of recorded visual-analogue-scale pain scores, available for a subset of participants (full period: 395 FM, 895 controls; pre-diagnosis window: 51 FM, 97 controls; medians [IQR] 7.0 [5.0–9.0] vs. 5.0 [2.0–8.0] and 8.0 [5.8–8.8] vs. 6.0 [3.0–8.0], respectively). Pain scores are shown because they bear on the interpretation of the pre-diagnosis window (Section 4.2).

## Data Availability

The data supporting the findings of this study are available from Leumit Health Services under licence and with permission; restrictions apply, and the data are not publicly available.

## Supplementary Materials

The following supporting information can be downloaded at https://www.mdpi.com/article/10.3390/jcdd13100474/s1: Table S1. Within-person variability of blood pressure and heart rate during the pre-diagnosis period; Table S2. Longitudinal time course of the principal cardiovascular measures in non-overlapping intervals relative to the index date; Table S3. Sensitivity of the pre-diagnosis differences to longer peri-diagnostic blackouts and to diagnostic-criteria era; Table S4. Restricted-cohort and multivariable confounder sensitivity analyses (pre-diagnosis window); Table S5. Supportive coded diagnoses in the pre-diagnosis window and blood-pressure-related medication use by class; Table S6. Detailed vital-sign distributions in fibromyalgia patients and matched controls; Table S7. Measurement intensity and robustness of the pre-diagnosis differences to measurement opportunity.

## Abbreviations

The following abbreviations are used in this manuscript:

Abbreviation	Meaning
ACR	American College of Rheumatology
ATC	Anatomical Therapeutic Chemical classification
BMI	Body mass index
BP	Blood pressure
CI	Confidence interval
CV	Coefficient of variation
EHR	Electronic health record
FM	Fibromyalgia
HDL	High-density lipoprotein
HR	Heart rate
ICD-9	International Classification of Diseases, 9th revision
IQR	Interquartile range
LHS	Leumit Health Services
OR	Odds ratio
SD	Standard deviation
SMD	Standardized mean difference
VAS	Visual analogue scale
