# Supplementary material for: Lower Blood Pressure Extremes Precede the Recorded Diagnosis of Fibromyalgia by up to Two Decades: A Population-Based Matched Case–Control Study
Source: J Cardiovasc Dev Dis. 2026 Sep 22;13(10):474. doi: 10.3390/jcdd13100474 (PMC13705504; doi:10.3390/jcdd13100474)
Supplement: Supplementary file 1 [file jcdd-13-00474-s001.zip › jcdd-4547874-supplementary.pdf]

Supplementary Materials

Lower Blood-Pressure Extremes Precede the Recorded Diagnosis of Fibromyalgia by Up to Two Decades: A Population-Based Matched Case–Control Study

Eli Magen, Suhail Aamar, Israel Magen, Eugene Merzon, Ilan Green, Avivit Golan-Cohen, Shlomo Vinker and Ariel Israel

Abbreviations: BP, blood pressure; CI, confidence interval; CV, coefficient of variation; DBP, diastolic blood pressure; FM, fibromyalgia; HR, heart rate; IQR, interquartile range; MAP, mean arterial pressure; OR, odds ratio; SBP, systolic blood pressure; SD, standard deviation; SMD, standardized mean difference (FM minus control). Negative differences indicate lower values in FM. Unless stated otherwise, the pre-diagnosis window covers up to 20 years before the index date with a 30-day blackout.

Table S1. Within-person variability of blood pressure and heart rate during the pre-diagnosis period.

Measure	n FM	n Control	FM mean	Control mean	SMD (95% CI)	p
Within-person SD of SBP (mmHg)	12,774	57,504	11.25	11.07	+0.038 (+0.019 to +0.058)	<0.001
Within-person CV of SBP	12,774	57,504	0.0927	0.0902	+0.077 (+0.058 to +0.096)	<0.001
Within-person SD of DBP (mmHg)	12,774	57,504	7.30	7.11	+0.075 (+0.056 to +0.094)	<0.001
Within-person CV of DBP	12,774	57,504	0.0977	0.0948	+0.087 (+0.068 to +0.106)	<0.001
Within-person SD of HR (bpm)	10,013	41,161	8.27	8.04	+0.057 (+0.035 to +0.079)	<0.001
Within-person CV of HR	10,013	41,161	0.1057	0.1028	+0.058 (+0.037 to +0.080)	<0.001

Calculated among participants with at least three eligible pre-diagnosis measurements.

Table S2. Longitudinal time course of the principal cardiovascular measures in non-overlapping intervals relative to the index date.

Interval relative to index date	Outcome	n FM	n Control	FM mean	Control mean	Difference (95% CI)	SMD	p
−20 to −15 y	Minimum SBP	2,909	13,014	106.66	109.37	−2.71 (−3.28 to −2.14)	−0.186	<0.001
−20 to −15 y	Minimum pulse pressure	2,909	13,014	35.48	37.11	−1.63 (−2.00 to −1.27)	−0.177	<0.001
−20 to −15 y	Maximum HR	1,917	8,248	81.15	80.48	+0.67 (0.13 to 1.21)	+0.062	0.015
−15 to −10 y	Minimum SBP	6,491	29,622	106.75	109.08	−2.33 (−2.69 to −1.96)	−0.165	<0.001
−15 to −10 y	Minimum pulse pressure	6,491	29,622	35.42	36.92	−1.50 (−1.74 to −1.26)	−0.164	<0.001
−15 to −10 y	Maximum HR	4,522	19,509	82.32	80.98	+1.34 (0.95 to 1.72)	+0.116	<0.001
−10 to −5 y	Minimum SBP	10,461	49,013	107.24	110.01	−2.77 (−3.05 to −2.49)	−0.201	<0.001
−10 to −5 y	Minimum pulse pressure	10,461	49,013	35.21	37.27	−2.06 (−2.25 to −1.87)	−0.223	<0.001
−10 to −5 y	Maximum HR	8,039	35,733	84.39	82.77	+1.63 (1.32 to 1.93)	+0.131	<0.001
−5 to −2 y	Minimum SBP	11,780	53,401	110.36	113.62	−3.26 (−3.53 to −2.98)	−0.226	<0.001
−5 to −2 y	Minimum pulse pressure	11,780	53,401	37.42	39.84	−2.41 (−2.61 to −2.22)	−0.243	<0.001
−5 to −2 y	Maximum HR	9,484	40,544	84.14	82.88	+1.26 (0.97 to 1.55)	+0.098	<0.001
−2 y to −30 d	Minimum SBP	12,468	52,411	113.60	116.79	−3.19 (−3.47 to −2.91)	−0.211	<0.001
−2 y to −30 d	Minimum pulse pressure	12,468	52,411	39.72	42.22	−2.49 (−2.70 to −2.29)	−0.231	<0.001
−2 y to −30 d	Maximum HR	9,992	40,283	83.59	81.90	+1.69 (1.41 to 1.97)	+0.132	<0.001

Interval relative to index date	Outcome	n FM	n Control	FM mean	Control mean	Difference (95% CI)	SMD	p
0 to 2 y	Minimum SBP	13,113	56,521	114.04	117.18	−3.14 (−3.42 to −2.86)	−0.204	<0.001
0 to 2 y	Minimum pulse pressure	13,113	56,521	39.93	42.55	−2.62 (−2.82 to −2.42)	−0.237	<0.001
0 to 2 y	Maximum HR	10,961	45,164	84.52	82.29	+2.23 (1.95 to 2.50)	+0.172	<0.001
2 to 5 y	Minimum SBP	12,810	57,324	113.28	116.62	−3.35 (−3.63 to −3.07)	−0.219	<0.001
2 to 5 y	Minimum pulse pressure	12,810	57,324	39.58	42.09	−2.51 (−2.71 to −2.30)	−0.226	<0.001
2 to 5 y	Maximum HR	11,429	49,125	85.30	83.42	+1.88 (1.61 to 2.16)	+0.143	<0.001
5 to 10 y	Minimum SBP	9,977	46,769	112.38	115.74	−3.37 (−3.68 to −3.06)	−0.223	<0.001
5 to 10 y	Minimum pulse pressure	9,977	46,769	39.11	41.42	−2.30 (−2.53 to −2.07)	−0.206	<0.001
5 to 10 y	Maximum HR	9,435	43,258	87.19	85.47	+1.72 (1.42 to 2.02)	+0.128	<0.001

Each participant contributes the per-patient minimum (or maximum) of the readings recorded within the interval. Minima within short intervals are higher than the lifetime minimum because fewer readings are sampled.

Table S3. Sensitivity of the pre-diagnosis differences to longer peri-diagnostic blackouts and to diagnostic-criteria era.

Panel A. Blackout length

Blackout	Outcome	Difference	95% CI	SMD	p
30 days	Minimum SBP	−3.27	−3.51 to −3.04	−0.239	<0.001
30 days	Minimum pulse pressure	−2.33	−2.49 to −2.18	−0.261	<0.001
30 days	Maximum HR	+2.21	1.95 to 2.47	+0.163	<0.001
90 days	Minimum SBP	−3.26	−3.49 to −3.02	−0.238	<0.001
90 days	Minimum pulse pressure	−2.30	−2.45 to −2.14	−0.257	<0.001
90 days	Maximum HR	+2.16	1.90 to 2.42	+0.160	<0.001
180 days	Minimum SBP	−3.28	−3.52 to −3.04	−0.241	<0.001
180 days	Minimum pulse pressure	−2.33	−2.49 to −2.17	−0.261	<0.001
180 days	Maximum HR	+2.14	1.87 to 2.40	+0.158	<0.001
365 days	Minimum SBP	−3.27	−3.51 to −3.03	−0.241	<0.001
365 days	Minimum pulse pressure	−2.32	−2.48 to −2.16	−0.261	<0.001
365 days	Maximum HR	+2.05	1.78 to 2.31	+0.153	<0.001

Panel B. Diagnostic-criteria era (year of index diagnosis)

Era	Outcome	n FM	n Control	Difference	95% CI	SMD	p
<2010 (ACR 1990)	Minimum SBP	2,484	11,189	−4.32	−4.95 to −3.68	−0.287	<0.001
<2010 (ACR 1990)	Minimum pulse pressure	2,484	11,189	−2.93	−3.35 to −2.52	−0.296	<0.001
<2010 (ACR 1990)	Maximum HR	1,783	7,542	+1.37	0.80 to 1.94	+0.127	<0.001
≥2010 (ACR 2010/2011 and later)	Minimum SBP	11,747	57,318	−3.17	−3.41 to −2.92	−0.245	<0.001
≥2010 (ACR 2010/2011 and later)	Minimum pulse pressure	11,747	57,318	−2.28	−2.44 to −2.12	−0.268	<0.001
≥2010 (ACR 2010/2011 and later)	Maximum HR	11,024	51,614	+2.45	2.17 to 2.73	+0.180	<0.001
2010–2015	Minimum SBP	4,034	19,253	−3.51	−3.93 to −3.08	−0.265	<0.001
2010–2015	Minimum pulse pressure	4,034	19,253	−2.52	−2.79 to −2.24	−0.294	<0.001

Era	Outcome	n FM	n Control	Difference	95% CI	SMD	p
2010–2015	Maximum HR	3,508	15,354	+1.92	1.48 to 2.37	+0.160	<0.001
≥2016 (ACR 2016 revision)	Minimum SBP	7,713	38,065	−3.02	−3.32 to −2.73	−0.238	<0.001
≥2016 (ACR 2016 revision)	Minimum pulse pressure	7,713	38,065	−2.18	−2.38 to −1.98	−0.259	<0.001
≥2016 (ACR 2016 revision)	Maximum HR	7,516	36,260	+2.88	2.54 to 3.23	+0.209	<0.001

Table S4. Restricted-cohort and multivariable confounder sensitivity analyses (pre-diagnosis window).

Panel A. Restricted cohort: no antihypertensive dispensing and no ischaemic heart disease, congestive heart failure, cerebrovascular disease, or diabetes before the pre-diagnosis cutoff

Outcome	n FM	n Control	FM mean	Control mean	Difference (95% CI)	SMD	p
Minimum SBP (mmHg)	8,107	43,634	100.78	103.76	−2.98 (−3.28 to −2.68)	−0.226	<0.001
Minimum DBP (mmHg)	8,107	43,634	62.62	64.38	−1.76 (−1.97 to −1.55)	−0.192	<0.001
Minimum MAP (mmHg)	8,107	43,634	76.24	78.32	−2.09 (−2.31 to −1.86)	−0.214	<0.001
Minimum pulse pressure (mmHg)	8,107	43,634	31.63	33.76	−2.12 (−2.32 to −1.92)	−0.242	<0.001
Maximum HR (bpm)	7,093	36,462	87.85	85.96	+1.89 (+1.55 to +2.24)	+0.142	<0.001

Panel B. Patient-level multivariable linear models

Model	Outcome	n	Adjusted FM − Control difference	95% CI	p
Primary	Minimum SBP (mmHg)	75,961	−1.44	−1.64 to −1.25	<0.001
Primary	Maximum HR (bpm)	68,285	+0.30	0.08 to 0.53	0.008
Extended cardiovascular	Minimum SBP (mmHg)	75,961	−1.18	−1.37 to −0.99	<0.001
Extended cardiovascular	Maximum HR (bpm)	68,285	+0.25	0.03 to 0.48	0.027

The primary model includes FM status, age, sex, BMI category and within-category BMI, smoking status, diabetes, dyslipidaemia, renal-function class (estimated glomerular filtration rate), any antihypertensive exposure, log-transformed number of readings of the outcome measure, and measurement rate. The extended cardiovascular model additionally includes socioeconomic-status category, ischaemic heart disease, congestive heart failure, cerebrovascular disease, chronic kidney disease, hypertension diagnosis, and beta-blocker exposure. Age and sex are the matching variables. n is the number of participants with complete covariate data.

Table S5. Supportive coded diagnoses in the pre-diagnosis window and blood-pressure-related medication use by class.

Panel A. Coded hypotension-related, autonomic, syncopal, and tachycardic diagnoses recorded in the pre-diagnosis window

Diagnosis (ICD-9 code)	FM, n (%)	Control, n (%)	OR (95% CI)	p	FDR-adjusted p
Orthostatic hypotension (458.0)	79 (0.50%)	214 (0.27%)	1.85 (1.41–2.41)	<0.001	<0.001
Chronic hypotension (458.1)	44 (0.28%)	65 (0.08%)	3.39 (2.26–5.05)	<0.001	<0.001
Hypotension, unspecified (458.9)	357 (2.25%)	1,006 (1.27%)	1.79 (1.58–2.03)	<0.001	<0.001
Autonomic disorder/neuropathy (337.0, 337.00, 337.90)	67 (0.42%)	70 (0.09%)	4.80 (3.38–6.81)	<0.001	<0.001
Syncope/presyncope (780.2, 780.22, 780.23)	1,813 (11.42%)	5,105 (6.43%)	1.88 (1.77–1.99)	<0.001	<0.001
Tachycardia, unspecified (785.0)	444 (2.80%)	1,381 (1.74%)	1.63 (1.45–1.81)	<0.001	<0.001

These are coded clinical diagnoses recorded in routine care and are presented as supportive evidence only; they are not substitutes for standardized orthostatic testing, tilt-table testing, or heart-rate-variability measurement. Percentages are of the full cohort (15,869 FM; 79,345 controls). FDR, Benjamini–Hochberg false-discovery rate.

Panel B. Blood-pressure-related medication dispensing by class

Medication (ATC class)	Full period: FM n (%)	Full period: Control n (%)	Full period: OR (95% CI)	Pre-dx: FM n (%)	Pre-dx: Control n (%)	Pre-dx: OR (95% CI)
Midodrine	65 (0.41%)	92 (0.12%)	3.54 (2.54–4.92)	41 (0.26%)	34 (0.04%)	6.04 (3.74–9.82)
Fludrocortisone	22 (0.14%)	52 (0.07%)	2.12 (1.22–3.55)	17 (0.11%)	33 (0.04%)	2.58 (1.35–4.76)
Other antihypertensives (C02)	1,502 (9.46%)	5,789 (7.30%)	1.33 (1.25–1.41)	674 (4.25%)	2,621 (3.30%)	1.30 (1.19–1.42)
Diuretics (C03)	3,932 (24.78%)	15,464 (19.49%)	1.36 (1.31–1.42)	2,083 (13.13%)	8,181 (10.31%)	1.31 (1.25–1.38)
Beta-blockers (C07)	6,025 (37.97%)	23,053 (29.05%)	1.49 (1.44–1.55)	3,525 (22.21%)	13,548 (17.07%)	1.39 (1.33–1.45)
Calcium-channel blockers (C08)	4,238 (26.71%)	18,087 (22.80%)	1.23 (1.19–1.28)	2,294 (14.46%)	9,785 (12.33%)	1.20 (1.14–1.26)
Renin–angiotensin-system blockers (C09)	6,520 (41.09%)	29,338 (36.98%)	1.19 (1.15–1.23)	3,690 (23.25%)	16,169 (20.38%)	1.18 (1.14–1.23)
Any antihypertensive (C02/C03/C07/C08/C09)	8,836 (55.68%)	36,766 (46.34%)	1.45 (1.41–1.51)	5,503 (34.68%)	22,687 (28.59%)	1.33 (1.28–1.37)

Pre-dx, dispensing from 20 years before the index date through 30 days before the index date. OR, odds ratio (FM vs. control).

Table S6. Detailed vital-sign distributions in fibromyalgia patients and matched controls.

Panel A. Full observation period

Measure	n (FM/Control)	FM mean ± SD	FM median [IQR]	Control mean ± SD	Control median [IQR]	Difference (95% CI)	SMD (95% CI)	p
Minimum SBP (mmHg)	15,837/78,440	99.13 ± 11.13	100.00 [90.00–107.00]	102.65 ± 12.30	101.00 [93.00–110.00]	−3.51 (−3.71 to −3.32)	−0.290 (−0.307 to −0.273)	<0.001
Minimum DBP (mmHg)	15,837/78,440	59.54 ± 7.98	60.00 [55.00–65.00]	61.72 ± 8.45	60.00 [57.00–68.00]	−2.19 (−2.32 to −2.05)	−0.261 (−0.278 to −0.244)	<0.001
Minimum MAP (mmHg)	15,837/78,440	74.41 ± 8.19	73.33 [69.67–80.00]	76.88 ± 8.94	76.67 [70.00–83.33]	−2.48 (−2.62 to −2.34)	−0.281 (−0.298 to −0.264)	<0.001
Minimum pulse pressure (mmHg)	15,837/78,440	29.33 ± 7.41	30.00 [25.00–34.00]	31.79 ± 8.03	30.00 [27.00–37.00]	−2.46 (−2.59 to −2.33)	−0.310 (−0.327 to −0.293)	<0.001
Maximum HR (bpm)	15,691/77,069	96.80 ± 13.47	96.00 [88.00–105.00]	93.85 ± 13.68	92.00 [84.00–102.00]	+2.95 (+2.72 to +3.19)	+0.216 (+0.199 to +0.234)	<0.001
Minimum body temperature (°C)	10,730/41,843	36.52 ± 0.61	36.40 [36.10–36.70]	36.67 ± 0.70	36.50 [36.20–36.90]	−0.15 (−0.17 to −0.14)	−0.225 (−0.246 to −0.203)	<0.001
Mean SBP (mmHg)	15,837/78,440	122.51 ± 12.45	122.00 [113.06–131.09]	123.61 ± 13.61	122.75 [113.46–132.80]	−1.11 (−1.32 to −0.89)	−0.082 (−0.100 to −0.065)	<0.001

Panel B. Pre-diagnosis window (up to 20 years before the index date; 30-day blackout)

Measure	n (FM/Control)	FM mean ± SD	FM median [IQR]	Control mean ± SD	Control median [IQR]	Difference (95% CI)	SMD (95% CI)	p
Minimum SBP (mmHg)	14,231/68,507	102.55 ± 12.88	100.00 [91.00–110.00]	105.82 ± 13.83	105.00 [96.00–115.00]	−3.27 (−3.51 to −3.04)	−0.239 (−0.258 to −0.221)	<0.001
Minimum DBP (mmHg)	14,231/68,507	63.12 ± 8.75	60.00 [59.00–70.00]	65.03 ± 9.23	65.00 [60.00–70.00]	−1.91 (−2.07 to −1.75)	−0.209 (−0.227 to −0.191)	<0.001
Minimum MAP (mmHg)	14,231/68,507	77.37 ± 9.36	76.67 [70.00–83.33]	79.64 ± 10.00	80.00 [72.67–86.33]	−2.27 (−2.45 to −2.10)	−0.230 (−0.248 to −0.212)	<0.001
Minimum pulse pressure (mmHg)	14,231/68,507	31.81 ± 8.53	30.00 [26.00–38.00]	34.14 ± 9.04	33.00 [30.00–40.00]	−2.33 (−2.49 to −2.18)	−0.261 (−0.279 to −0.242)	<0.001
Maximum HR (bpm)	12,807/59,156	89.51 ± 13.74	88.00 [80.00–98.00]	87.30 ± 13.50	85.00 [78.00–96.00]	+2.21 (+1.95 to +2.47)	+0.163 (+0.144 to +0.182)	<0.001

Measure	n (FM/Control)	FM mean ± SD	FM median [IQR]	Control mean ± SD	Control median [IQR]	Difference (95% CI)	SMD (95% CI)	p
Minimum body temperature (°C)	6,334/23,453	36.73 ± 0.73	36.60 [36.20–37.00]	36.88 ± 0.80	36.60 [36.30–37.20]	−0.15 (−0.17 to −0.12)	−0.186 (−0.213 to −0.158)	<0.001
Mean SBP (mmHg)	14,231/68,507	120.13 ± 12.92	119.12 [110.64–128.50]	121.09 ± 13.95	120.00 [110.88–130.00]	−0.96 (−1.19 to −0.72)	−0.069 (−0.087 to −0.051)	<0.001

Values are per-patient summaries. p-values are from two-sided Welch t-tests.

Table S7. Measurement intensity and robustness of the pre-diagnosis differences to measurement opportunity.

Analysis	Outcome / stratum	n FM	n Control	FM − Control difference	95% CI	SMD	p
Measurement intensity	BP readings per patient	15,869	79,345	+6.73	6.19 to 7.27	+0.248	<0.001
Measurement intensity	Encounters with a BP reading	15,869	79,345	+6.45	5.92 to 6.97	+0.245	<0.001
First 3 readings	Minimum SBP	12,774	57,504	−1.66	−1.94 to −1.38	−0.110	<0.001
First 3 readings	Maximum HR	10,013	41,161	+0.38	0.14 to 0.63	+0.034	0.002
First 5 readings	Minimum SBP	11,574	49,268	−1.86	−2.14 to −1.58	−0.130	<0.001
First 5 readings	Maximum HR	8,018	30,691	+0.64	0.35 to 0.93	+0.055	<0.001
First 10 readings	Minimum SBP	8,988	34,727	−2.05	−2.36 to −1.75	−0.152	<0.001
First 10 readings	Maximum HR	4,878	17,105	+1.04	0.65 to 1.43	+0.085	<0.001
BP-count stratum	2–5 readings, minimum SBP	2,468	16,735	−1.18	−1.73 to −0.63	−0.087	<0.001
BP-count stratum	6–10 readings, minimum SBP	2,417	13,173	−1.97	−2.48 to −1.46	−0.160	<0.001
BP-count stratum	11–20 readings, minimum SBP	3,173	14,548	−1.94	−2.38 to −1.49	−0.159	<0.001
BP-count stratum	21–50 readings, minimum SBP	3,714	13,298	−2.60	−3.02 to −2.18	−0.217	<0.001
BP-count stratum	>50 readings, minimum SBP	1,687	4,749	−2.83	−3.42 to −2.23	−0.263	<0.001
Adjusted for log measurement count and rate	Minimum SBP	—	—	−1.95	−2.19 to −1.72	—	<0.001
Adjusted for log measurement count and rate	Maximum HR	—	—	+0.62	0.39 to 0.86	—	<0.001
Patient-level lower-tail sensitivity	10th percentile of SBP	14,231	68,507	−2.01	−2.23 to −1.80	−0.158	<0.001

The first-n analyses equalise measurement opportunity by limiting each participant to the first n chronological pre-diagnosis readings. The adjusted models are patient-level linear models including log-transformed measurement count and measurement rate (readings per year of observation). Measurement-count strata are defined by total pre-diagnosis BP readings; participants with a single reading were not analysed.
